# Efficacy, Dosage, and Duration of Action of Branched Chain Amino Acid Therapy for Traumatic Brain Injury

**DOI:** 10.3389/fneur.2015.00073

**Published:** 2015-03-30

**Authors:** Jaclynn A. Elkind, Miranda M. Lim, Brian N. Johnson, Chris P. Palmer, Brendan J. Putnam, Matthew P. Kirschen, Akiva S. Cohen

**Affiliations:** ^1^Division of Neurology, Children’s Hospital of Philadelphia, Philadelphia, PA, USA; ^2^Sleep Disorders Laboratory, Division of Hospital and Specialty Medicine, Veterans Affairs Portland Healthcare System, Portland, OR, USA; ^3^Department of Medicine, Oregon Health & Science University, Portland, OR, USA; ^4^Department of Behavioral Neuroscience, Oregon Health & Science University, Portland, OR, USA; ^5^Department of Neuroscience, Perelman School of Medicine, University of Pennsylvania, Philadelphia, PA, USA; ^6^Department of Anesthesia and Critical Care, Children’s Hospital of Philadelphia, Philadelphia, PA, USA; ^7^Department of Pediatrics, Perelman School of Medicine, University of Pennsylvania, Philadelphia, PA, USA

**Keywords:** traumatic brain injury, branched chain amino acids, hippocampus, fear conditioning, network excitability

## Abstract

Traumatic brain injury (TBI) results in long-lasting cognitive impairments for which there is currently no accepted treatment. A well-established mouse model of mild to moderate TBI, lateral fluid percussion injury (FPI), shows changes in network excitability in the hippocampus including a decrease in net synaptic efficacy in area CA1 and an increase in net synaptic efficacy in dentate gyrus. Previous studies identified a novel therapy consisting of branched chain amino acids (BCAAs), which restored normal mouse hippocampal responses and ameliorated cognitive impairment following FPI. However, the optimal BCAA dose and length of treatment needed to improve cognitive recovery is unknown. In the current study, mice underwent FPI then consumed 100 mM BCAA supplemented water *ad libitum* for 2, 3, 4, 5, and 10 days. BCAA therapy ameliorated cognitive impairment at 5 and 10 days duration. Neither BCAA supplementation at 50 mM nor BCAAs when dosed 5 days on then 5 days off was sufficient to ameliorate cognitive impairment. These results suggest that brain injury causes alterations in hippocampal function, which underlie and contribute to hippocampal cognitive impairment, which are reversible with at least 5 days of BCAA treatment, and that sustaining this effect is dependent on continuous treatment. Our findings have profound implications for the clinical investigation of TBI therapy.

## Introduction

Traumatic brain injury (TBI) is a worldwide problem and a major cause of disability in US. The annual incidence of TBI is over 1.2 million people and approximately 50,000 TBI-related deaths occur each year (1). Costs from TBI exceed more than $37 billion per year in US alone, due in part to the over 5 million people with chronic disabilities resulting from TBI (2, 3). There are currently no accepted therapies for the cognitive deficits attributed to TBI (4, 5).

A well-established mouse model of mild to moderate TBI, lateral fluid percussion injury (FPI), produces deficits similar to those reported after human TBI, including memory deficits, pathological gliosis, and ionic perturbations (6, 7). The hippocampus, a brain structure vital to learning and memory, is particularly vulnerable to TBI. Optimal hippocampal function requires a delicate balance between neuronal excitation and inhibition. This balance determines net synaptic efficacy in the hippocampus, which is profoundly and chronically altered after FPI in a region-specific manner (8–12). In the CA1 region of the hippocampus, FPI causes a decrease in network excitability, while in the dentate gyrus network excitability is increased (8).

The conditioned fear response (CFR), a widely accepted behavioral test of hippocampal function (13), is measured by the extent of “freezing” (animal remains motionless) when the animal is returned to the location in which it previously received an aversive foot shock. Mild to moderate FPI causes deficits in fear conditioning, which were shown previously to reflect hippocampal dysfunction (14).

Recent studies in our laboratory demonstrated that hippocampal concentrations of the branched chain amino acids (BCAAs: leucine, isoleucine, and valine) are reduced after TBI and treatment with these amino acids restored cognitive function to levels comparable to non-injured mice (15). Furthermore, BCAA therapy mitigated injury-induced deficits in maintaining wakefulness and also restored electroencephalography (EEG) rhythms associated with normal wakefulness, in mice after TBI (16). These animal data are consistent with studies in humans demonstrating improved disability rating scale scores after treatment with intravenous BCAAs in adults recovering from severe TBI (17, 18). BCAAs are essential amino acids (i.e., they are acquired solely through dietary intake) and are precursors for the *de novo* synthesis of the neurotransmitters glutamate and GABA (19, 20). They are also a major source of intermediates for the citric acid cycle, and BCAA supplementation been used to treat diseases associated with alterations in their metabolism [reviewed in Ref. (17, 21)]. Plasma and hippocampal levels of the BCAAs are reduced after TBI (15, 22, 23), due perhaps in part to the metabolic disturbances associated with the brain injury. While full details of the mechanism by which BCAAs act is not currently known, the BCAAs may improve hippocampal function after TBI by restoring releasable pools of glutamate and GABA. Consistent with this hypothesis, BCAA treatment restored normal hippocampal stimulus-evoked responses *in vitro* when BCAAs were bath applied to hippocampal slices prepared from injured animals, and also when hippocampal slices were prepared from brain-injured mice that had been treated with BCAAs in their drinking water [100 mM BCAA supplemented drinking water for 5 days (15)]. The current experiments were designed to (1) determine the minimum treatment time and dosage of BCAA therapy necessary to restore normal behavior, and (2) determine if the functional improvements produced by BCAA therapy are sustained after therapy is stopped.

## Materials and Methods

### Animals

All experiments were performed on 5- to 7-week-old, 20–25 g, male C57BL/J6 mice (Jackson Laboratory). The animals were housed in a room that was maintained at an ambient temperature of 23 ± 1°C with a relative humidity of 25 ± 5% and that was on an automatically controlled 12-h light/12-h dark cycle (light on at 07:00 hours, illumination intensity ≈100 lux). The animals had free access to food and water. Every effort was made to minimize the number of animals used and any pain and discomfort experienced by the mice. Animal experiments were performed in accordance with the guidelines published in the National Institutes of Health Guide for the Care and Use of Laboratory Animals and were approved by the Children’s Hospital of Philadelphia Animal Care and Use Committee in accordance with international guidelines on the ethical use of animals.

### Fluid percussion injury

Mice were anesthetized using a combination of ketamine (2.6 mg/kg) and xylazine (1.6 mg/kg) and placed in a stereotaxic frame (Stoelting). A midline incision in the scalp was made and a 3 mm diameter, 1 mm height disk was glued to the skull using the tissue glue, Vetbond. Between lambda and bregma, lateral to the sagittal suture on the right side, a trephine (3 mm diameter) was used to perform a craniectomy. A luer-loc needle hub with a 3 mm diameter was secured to the skull around the opening using Loctite glue and dental cement. The luer-loc was filled with saline and capped for 24 h. The next day, animals underwent lateral FPI. They were anesthetized using 2% isoflurane, the cap was removed and the luer-loc was filled with saline to ensure the fluid pulse was consistent and continuous, and free from air bubbles. The luer-loc was then secured to the FPI device (Department of Biomedical Engineering, Virginia Commonwealth University, Richmond, VA, USA) and a fluid pulse of 20 ms was administered. The injury pressure was measured via an oscilloscope attached to the injury device and for all animals was in a range of 1.8–2.1 atm. After recovering on a heating pad, the luer-loc was removed and the scalp was sutured. Mice were returned to their home cage for recovery. Animals were allowed to recover for 48 h prior to initiating treatment.

### Study design

**Figure 1:**
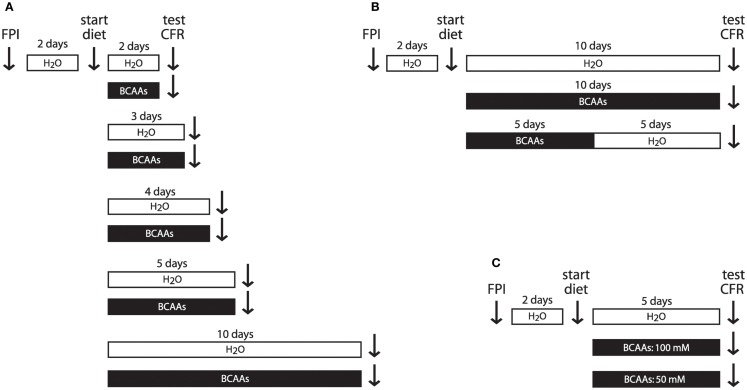
**Experimental timeline**. **(A)** To determine the effect of treatment duration on cognitive recovery, injured mice received BCAA supplemented drinking water or untreated water for varying lengths of time. Vertical arrows indicate times for injury, treatment onset, and conditioned fear testing. **(B)** To determine the duration of action of BCAA treatment, mice receiving 10 consecutive days of BCAA therapy were compared to mice receiving 5 days of BCAA supplemented water followed by 5 days of untreated water. **(C)** To determine the effective concentration of BCAA supplemented water, mice consumed 5 days of either 100 or 50 mM BCAA supplemented water or untreated water. Abbreviations: FPI, fluid percussion injury; CFR, conditioned fear response, BCAAs, branched chain amino acids.

Mice were housed individually and in all experiments except the gavage administration experiments drank *ad libitum* either BCAA supplemented water or untreated water. In the time course experiments, mice were treated with BCAAs for 2, 3, 4, 5, or 10 days as indicated (Figure 1A). In the gavage administration experiments, mice received oral gavage once daily with a solution containing BCAAs, or untreated water, for 3 or 5 days in order to compare *ad libitum* consumption of BCAAs with oral gavage delivery of a more precisely delivered amount. In the duration of action experiments, mice received 100 mM BCAA supplemented drinking water for 5 days, as in the initial time course experiments, but then received 5 days of untreated drinking water (Figure 1B) prior to behavioral testing. In the supplement concentration experiments, mice received drinking water that was either 50 or 100 mM in BCAAs (Figure 1C).

### BCAA treatment

Drinking water in the treatment groups was supplemented with 100 or 50 mM concentrations of each of leucine, isoleucine, and valine and consumed *ad libitum* (time course, duration of action, and supplement concentration experiments). Control treatment consisted of drinking untreated tap water. The amount of drinking water remaining in the bottle was measured each day, and fresh BCAA supplemented water (treatment groups) or fresh untreated water (control groups) was replaced each week. Mice drank on average 3–5 mL of solution per day and it has been previously shown that BCAAs do not affect body weight (15). A similar dose of BCAA’s (0.26 g BCAAs in equimolar amounts/kg of mouse weight) was delivered via oral gavage once daily (gavage volume for a typical 25 g mouse was 170 μL) per treatment day in the gavage administration experiments.

### Conditioned fear response

After recovery from FPI each animal was handled daily for 3 min over the 3 days leading up to fear conditioning (except for the animals tested at day 2, which were handled for only 2 days prior to testing). On day prior to CFR testing, mice were placed in the fear conditioning chamber (Med Associates, St. Albans, VT, USA) for a total of 3 min. After 2 min and 28 s in the chamber, animals received a 2-s shock of 1.05 mA intensity via the metal bars comprising the floor of the box. Animals remained in the box for an additional 30 s before being removed and placed back into the home cage. The box was cleaned with 70% ethanol in between the training of each animal. Twenty-four hours later, mice were returned to the fear-conditioning box for a total of 5 min. Observation of freezing behavior occurred every 5 s for the entire 5 min, for a total of 60 observations. Freezing percentage was calculated by dividing the number of observed instances of freezing by the total number of observations. Thus, a lower freezing percentage is indicative of impaired contextual memory.

### Statistical procedures

Data collection and analysis were performed blinded to experimental group. Statistical analyses were performed using GraphPad Prism (San Diego, CA, USA). Data were tested to ensure normality, and subsequently analyzed using either two-tailed Student’s *t*-tests, two-way ANOVA with Bonferroni *post hoc* tests, or one-way ANOVA followed by Dunnett’s *post hoc* tests if the *F* values reached statistical significance (*P* < 0.05). Statistical significance was defined at the *P* < 0.05 confidence level. Power analyses were performed to determine group sizes. In previous studies, the response within each subject group was normally distributed with a SD of ≈25%. If the true difference in the experimental and control means is ≈50%, we calculated the need to study at least five subjects per group to be able to reject the null hypothesis that the population means of the experimental and control groups are equal with probability (power) 0.8. The Type I error probability associated with this test of this null hypothesis is 0.05. All data are presented as group means ± SEM.

## Results

### BCAA therapy restores conditioned fear responses in brain-injured mice

Previous research from our lab demonstrated that freezing behavior in the CFR is impaired after mild to moderate FPI, and restored with 5 days of BCAA therapy (15). However, the minimum required treatment time, the minimum effective dose, and the duration of action of BCAA treatment are still unknown. As all of these values are necessary for the development of a translatable therapeutic regimen, they were determined in the following experiments.

**Figure 2:**
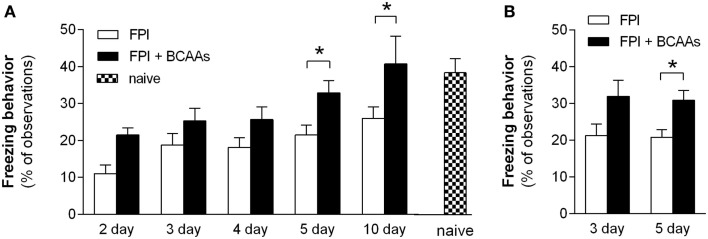
**Time course of BCAA treatment required to restore conditioned fear response**. **(A)** BCAA supplemented water (100 mM, black bars) or plain water (open bars) were offered *ad libitum* to brain-injured mice for 2, 3, 4, 5, or 10 days. Conditioned fear responses, as measured by the percentage of observations in which the animal demonstrated freezing behavior, were recorded for each animal. All animals in all treatment groups demonstrated at least some freezing, and the average level of freezing was calculated for each group. Freezing was restored to normal levels at the 5- and 10-day time points (*P < 0.05). Conditioned fear responses in naïve animals were not significantly different from conditioned fear responses in either the 5- or 10-day BCAA treated groups. **(B)** Water containing BCAAs (0.26 g/kg) or untreated water was administered via oral gavage once daily for 3 or 5 days. Significant improvement in freezing behavior was observed following five consecutive days of gavage BCAA treatment (*P < 0.05), but not after 3 days of treatment, and 5-day gavage results were not significantly different from the 5-day supplemented water results. Abbreviations: FPI, fluid percussion injury; BCAAs, branched chain amino acids.

In the time course experiments, mice consumed BCAA supplemented drinking water or untreated water *ad libitum* for 2, 3, 4, 5, or 10 consecutive days before undergoing CFR testing (Figure 1A). All animals tested demonstrated freezing. Freezing in individual animals ranged from 6.7% (measured 2 days after injury, in the untreated group) to 68.3% (measured 10 days after injury, in the 10-day BCAA treated group). As a group, injured mice that received BCAA treatment showed a significantly greater freezing response on average compared to untreated FPI mice when treatment was delivered for 5 or 10 consecutive days (Figure 2A; *F* = 18.12, *P* < 0.0001, two-way ANOVA; *P < 0.05, Bonferroni *post hoc* tests; untreated n’s, 2 through 10 days: 4, 8, 7, 16, 12; treated n’s, 2 through 10 days: 8, 7, 8, 15, 5). Although BCAA treatment also increased freezing in mice treated for 2, 3, and 4 days, those improvements were not statistically significant. These results suggest that BCAA supplementation improves cognitive recovery if consumed for at least five consecutive days after injury.

We also measured fear conditioning in a set of naïve animals in order to assess the extent of the cognitive recovery in the BCAA treated groups, which showed significant improvement (Figure 2A). Freezing in naïve animals was 38.8 ± 3.8% (*n* = 10), and was not significantly different from freezing in the 5-day (*n* = 16) or 10-day (*n* = 5) BCAA treated groups (*P* = 0.394 for naive, 5- and 10-day BCAA treated mice, one-way ANOVA). In summary, treatment with BCAAs for 5 or 10 days restored freezing behavior in injured mice to normal levels, indicating complete recovery of fear conditioning after treatment with BCAAs.

To compare the efficacy of treatment with BCAA supplemented drinking water to treatment with a more precisely known dose of BCAAs, mice were given BCAAs via oral gavage. Mice underwent FPI surgery as above, and then 2 days following injury began daily treatment with BCAAs via oral gavage for 3 or 5 days at a dose chosen to replicate our estimate of the dose received via BCAA supplemented drinking water (0.26 g BCAAs/kg of mouse weight). Injured mice receiving BCAAs via oral gavage for 5 days showed significantly greater freezing than FPI mice receiving untreated water via oral gavage (Figure 2B; *t* = 2.752, *P* = 0.0175, Student’s two-tailed *t*-test). Freezing in the 5-day gavage mice was not significantly different from freezing from the 5-day BCAA supplemented drinking water mice (*t* = 0.419, *P* = 0.679, Student’s two-tailed *t*-test; 5-day gavage: 32.9 ± 3.2%, *n* = 15; 5-day supplemented water: 30.8 ± 2.7%, *n* = 8). The gavage results are consistent with the *ad libitum* supplementation findings that BCAA therapy at these doses requires at least 5 days of continuous administration in order to significantly improve cognitive recovery, and those 3 days of therapy are not sufficient. The gavage results also suggest that treatment with a BCAA dose of 0.26 g/kg is equivalent to treatment with *ad libitum* 100 mM BCAA supplemented drinking water.

**Figure 3:**
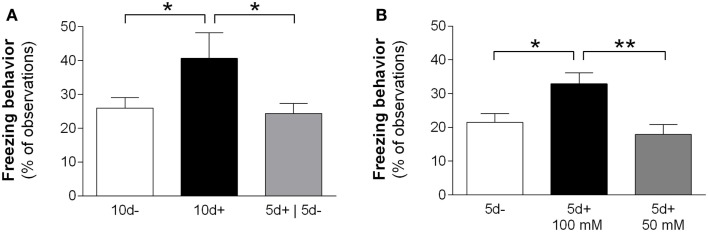
**Dosage effects of BCAA treatment**. **(A)** BCAA supplemented water administered *ad libitum* for 10 days after injury produced significant improvement in conditioned fear responses compared to brain-injured mice given untreated water for 10 days. Mice receiving BCAA supplemented water for 5 days, then untreated water for 5 days, did not show significant improvements in conditioned fear responses and were not significantly different from injured mice receiving no treatment (*P < 0.05). These results suggest that BCAAs need to be continuously administered in order to maintain cognitive improvement after brain injury. **(B)** BCAA supplemented water administered *ad libitum* at 50 and 100 mM for 5 days. Significant cognitive improvement in brain-injured mice was produced with 100 mM BCAA supplemented water, but not with 50 mM BCAA supplemented water (*P < 0.05 and **P < 0.01). Abbreviations describing treatment received by injured animals prior to testing: 5d− and 10d−, drinking water without BCAAs for 5 and 10 days, respectively; 10d+, 100 mM BCAA supplemented drinking water for 10 days; 5d+|5d−, 100 mM BCAA supplemented drinking water for 5 days, followed by 5 days with plain water; 5d−, no treatment for 5 days; 5d+ 100 mM, 100 mM BCAA supplemented drinking water for 5 days; 5d+ 50 mM, 50 mM BCAA supplemented drinking water for 5 days.

To examine the duration of action of BCAA treatment, a separate cohort of mice were given 100 mM BCAA supplemented water for 5 days followed by for 5 days of untreated drinking water (i.e., 5d+|5d− treatment) (Figure 1B). Freezing in the TBI 5d+|5d− mice was significantly lower than freezing in TBI 10d+ mice, and was not significantly different from freezing in the untreated TBI 10d− mice (Figure 3A; *F* = 3.67, *P* = 0.0421, TBI 5d+|5d− *n* = 8, TBI 10d+ *n* = 5, TBI 10d− *n* = 8, one-way ANOVA; *P* < 0.05, Dunnett’s *post hoc* tests). In other words, the TBI 5d+|5d− group reverted back to the injured phenotype. These data suggest that BCAA treatment lasting only 5 days does not provide long-lasting benefits, and that ongoing BCAA treatment is needed to maintain optimal hippocampal function.

To determine if a lower concentration of BCAA supplemented water could also ameliorate cognitive impairment after injury, a separate group of animals received only 50 mM of BCAAs *ad libitum* for 5 days, and were compared to the injured animals that received 100 mM BCAA supplemented drinking water for 5 days (Figure 1C). Injured mice in the 50 mM group showed significantly less freezing than mice in the 100 mM group, and were not significantly different from untreated injured mice (Figure 3B; *F* = 6.295, *P* = 0.0045, injured untreated *n* = 16, 100 mM *n* = 15, 50 mM *n* = 8, one-way ANOVA, Tukey’s *post hoc* tests). These data indicate that for treatment of injured mice with BCAA supplemented drinking water, 50 mM BCAAs is not a high enough concentration to restore normal fear conditioning.

Overall, the results above show that BCAA treatment restores normal hippocampal functioning after brain injury as assessed by contextual fear conditioning. Although treatment with BCAAs for 5 days does not permanently restore normal hippocampal function, continuous BCAA treatment for at least 5 days does restore and maintain normal hippocampal responses.

## Discussion

Previous data from our laboratory demonstrated that BCAA supplementation restores contextual fear conditioning and normal hippocampal network excitability after mild to moderate TBI (15). The current study includes additional experiments that explore the dependence of BCAA therapy on the time course, method and amount of BCAA administration, and also the duration of action of BCAA therapy.

Our data establish that BCAA therapy is required for at least five consecutive days at a dose of either 100 mM in *ad libitum* drinking water or 0.26 g/kg via oral gavage, to restore normal fear conditioning. Furthermore, stopping therapy, after 5 days, results in a functional relapse to levels seen in untreated injured animals. These results suggest the persistence of a functional deficit after TBI, which ongoing BCAA supplementation can successfully treat.

Our results complement and expand those of Aquilani et al. who found modest reduction in levels of disability in severely brain-injured patients after intravenous administration of BCAAs (17, 18). Enteral administration of BCAA therapy confers several benefits including slower BCAA uptake (absorption), which does not saturate CNS BCAA transporters on the blood–brain barrier. Dietary therapy is also advantageous in that patients recovering from TBI, and mild TBI in particular, do not require hospitalization and intravenous access for BCAA administration.

This enduring cognitive dysfunction may be due to alterations in hippocampal metabolism after TBI, with an associated reduction in BCAA concentrations (15). Approximately 50% of brain glutamate and 40% of the releasable synaptic glutamate contain BCAA-derived nitrogen (20). It is possible that BCAA supplementation improves hippocampal functioning by restoring pools of releasable vesicular glutamate and GABA. Measurements of glutamate and GABA in injured brain have not been able to distinguish synaptic pools of glutamate and GABA from the much larger and independent metabolic pools of glutamate and GABA (15). A recent report using cultured cerebellar neurons, however, suggests that at least one of the BCAAs, valine, does indeed become incorporated into the glutamate found in synaptic vesicles (24). Our results indicate that BCAAs must be consumed continuously for at least 5 days after injury to restore cognitive function, and that ongoing supplementation is necessary to maintain this cognitive recovery. These findings are consistent with the hypothesis that replenishment of synaptically releasable glutamate and GABA may be the primary mechanism by which normal hippocampal functioning is restored.

In addition to its effects in the hippocampus, BCAA therapy may have widespread effects in other brain networks as well, such as those involved in sleep and wakefulness. A recent study from our laboratory demonstrated that BCAA supplementation improved wakefulness after FPI, acting at least in part via orexin neurons in the lateral hypothalamus (16). BCAA therapy also restored EEG rhythms indicative of normal wakefulness, suggesting that BCAA effects are likely not restricted to the hippocampus alone (16).

Our results from this preclinical mouse model suggest that BCAA therapy has great potential as a treatment for the neurocognitive deficits from TBI. Such therapies are much needed, as there are currently no pharmacological interventions available. Advantages of this therapy include enteral and parenteral administration and being able to commence therapy 48 h after brain injury, thereby qualifying BCAA treatment as a neurorestorative rather than a neuroprotective therapy. Neurorestorative therapies have a significant advantage because they can be offered *after* an individual has sustained a TBI.

Decreased BCAA concentrations have also been reported in humans after TBI and parenteral BCAA administration improved disability outcomes after severe TBI (17, 22, 23). BCAAs have been used therapeutically for a variety of other neurological conditions and overall well tolerated and associated with minimal to no side effects (21, 25, 26). In summary, BCAAs are a potential neurorestorative therapy for TBI and these data could inform the development of human clinical trials.

## Conflict of Interest Statement

Akiva S. Cohen and the Children’s Hospital of Philadelphia hold a provisional patent, which includes the use of branched chain amino acids as a therapeutic intervention for traumatic brain injury: U.S. Provisional Patent Application Nos. 61/883,526 and 61/812,352, filed under the title “Compositions and methods for the treatment of brain injury.” This material is partially the result of work supported with resources at the VA Portland Health Care System. The contents do not represent the views of the U.S. Department of Veterans Affairs or the United States Government. The other co-authors declare that the research was conducted in the absence of any commercial or financial relationships that could be construed as a potential conflict of interest.
